# Response to the Letter to the Editor Entitled “Keeping Clinical CKM Staging Distinct From Biological Enrichment”

**DOI:** 10.1016/j.ekir.2026.107096

**Published:** 2026-09-22

**Authors:** William R. Marshall

**Affiliations:** 1 Division of Cardiovascular Sciences, University of Manchester, Manchester, UK; 2 Donal O’Donoghue Renal Research Centre, Northern Care Alliance NHS Foundation Trust, Salford, UK

The Author Replies:

We thank Peng and Wu^1^ for their careful reading of our review.^2^ They are correct about the cardiovascular-kidney-metabolic stage 4 definition. Our table defines stage 4 as clinical cardiovascular disease or kidney failure; kidney failure subdivides stage 4 rather than defining it independently.^3^^,^^4^ As written, the table upstages patients with kidney failure and no clinical cardiovascular disease. Stage 3 should have read pre-HF. Our stage 4 row already lists symptomatic heart failure alongside ischemic heart disease, stroke, and peripheral vascular disease, and the adjacent left ventricular hypertrophy entry denotes the same preclinical stage.

Regarding MASLD we accepted, in reply to an earlier letter on this review,^5^ that we had allowed the diagnosis alone to modify cardiovascular-kidney-metabolic stage 1 and that fibrosis burden should determine how much weight the modifier carries. Peng and Wu are correct that fibrosis-informed phenotyping is the variable that matters and that testing for incremental value must precede any upstaging role. The measurement follows: fibrosis stage, determined by the Fibrosis-4 index or elastography, entered as an increment to a model chosen for the endpoint, such as PREVENT for cardiovascular events, and assessed based on calibration and net benefit rather than discrimination alone.

The prognostic-versus-predictive distinction applies to conventional markers too. Enrichment by natriuretic peptides or albuminuria raises event rates and improves trial efficiency without establishing that the treatment effect differs; only a treatment-by-biomarker interaction does that. Figure 2 and the accompanying text move from an elevated molecular signal to earlier cardioprotective therapy without that step. They also list trial enrollment alongside therapy, although enrollment is how the therapy question gets answered.

The MASLD and prognostic-versus-predictive questions resolve the same way, by the recommendation we made in that earlier reply^5^: serial biobanking embedded in cardiovascular-kidney-metabolic outcome trials, which already adjudicate endpoints under standardized follow-up. That provides the incremental-value evidence a staging claim requires. Randomization additionally allows prespecified testing of whether benefit varies with the biomarker, which is what a predictive claim needs.

## Disclosure

The authors declared no competing interests.

### Funding

No specific funding was received.

## Data Availability Statement

No new data were generated or analyzed in this work.

## Author Contributions

WRM drafted and revised the manuscript.

### Declaration of AI and AI-Assisted Technologies in the Writing Process

No generative AI tools were used in the preparation of this manuscript.
